# Insulin Requirements During Severe COVID-19 Were Relatively Low in Japanese Patients With Type 2 Diabetes: Two Case Reports

**DOI:** 10.7759/cureus.47654

**Published:** 2023-10-25

**Authors:** Junpei Yamamoto, Hironobu Takahashi, Takaharu Saito, Yuri Yamamoto, Koudai Takahashi, Koji Itakura, Makoto Kobayashi, Ryotaro Igusa, Takafumi Kobayashi, Masakazu Ichinose, Masahiro Usui

**Affiliations:** 1 Division of Metabolism and Diabetes, Osaki Citizen Hospital, Osaki, JPN; 2 Division of Respiratory Medicine, Osaki Citizen Hospital, Osaki, JPN; 3 Division of Anesthesiology, Osaki Citizen Hospital, Osaki, JPN

**Keywords:** il-6 receptor antibody, ethnic differences, type 2 diabetes, insulin requirements, covid-19

## Abstract

The global coronavirus disease 2019 (COVID-19) pandemic has caused myriad adverse effects on the pathology of other diseases. Numerous studies on COVID-19 have reported that, in patients with type 2 diabetes mellitus (T2DM) who have contracted severe COVID-19, glucose metabolism is exacerbated by multiple factors, such as severe inflammation, beta-cell dysfunction caused by the SARS-CoV-2 infection itself, corticosteroid therapy, vasopressor administration, and enteral or parenteral nutrition. Very high doses of insulin are often required in the acute phase of such patients; however, the factors that affect insulin requirements and to what extent remain unclear.

A 50-year-old Japanese woman and a 67-year-old Japanese man, both with T2DM and obesity, were admitted to our hospital with severe COVID-19. Both patients required mechanical ventilation and were treated with dexamethasone and tocilizumab, an interleukin-6 (IL-6) receptor monoclonal antibody. Subcutaneous insulin injections failed to control the patients’ hyperglycemia, requiring up to 1.83 and 1.81 units/kg/day of intravenous insulin, respectively. Insulin requirements were rapidly decreased with improvement of the respiratory condition, termination of dexamethasone, and discontinuation of tube feeding. Both patients were discharged with oral antidiabetic agents alone.

We experienced two Japanese patients who achieved satisfactory glycemic control with a lower intravenous insulin dose than previous reports. Comparing the clinical factors with the previous literature, ethnic differences in insulin sensitivity and the administration of IL-6 receptor antibodies may have been related to the relatively low insulin requirements.

## Introduction

Since December 2019, the coronavirus disease 2019 (COVID-19) pandemic has significantly impacted global public health. Pre-existing diabetes mellitus has been reported as a determinant of the severity and mortality of COVID-19 patients [1]. Diabetes mellitus is also associated with poor outcomes in hospitalized COVID-19 patients [2]. Recent studies have also shown that both hyperglycemia and hypoglycemia [3], or glycemic fluctuations in the initial phase of hospitalization [4], are associated with poor outcomes in patients with COVID-19.

In COVID-19 patients, insulin resistance caused by severe inflammation [5] or beta-cell dysfunction induced by SARS-CoV2 infection itself [6] has been reported to disturb glucose tolerance. Moreover, other studies have suggested that the serum levels of inflammatory biomarkers such as C-reactive protein, D-dimer, interleukin-6 (IL-6), serum ferritin, and coagulation index are higher in COVID-19 patients with diabetes mellitus than in those without diabetes mellitus. These findings indicate that COVID-19 patients with diabetes mellitus are more vulnerable to inflammatory storms [7]. Furthermore, several medical treatments, such as the administration of corticosteroids, vasopressors, and tube feeding, further deteriorate glucose metabolism in critically ill patients. Hyperglycemia caused by these mechanisms is much more serious in patients with pre-existing diabetes. Indeed, severe COVID-19 patients with coexisting diabetes mellitus have been shown to require much higher insulin doses [8,9]. Clarifying the changes in insulin requirements and optimizing insulin doses in the acute phase of severe COVID-19 with diabetes mellitus are significant clinical issues.

Because East Asians are known to have insufficient insulin secretion capacity compared to Caucasians [10], the approach to optimizing the insulin dosage for achieving glycemic control in East Asian COVID-19 patients may differ from previous reports from Western countries. Herein, we report two cases of obese Japanese patients with type 2 diabetes mellitus (T2DM) who developed severe COVID-19.

## Case presentation

Case 1

**Figure 1:**
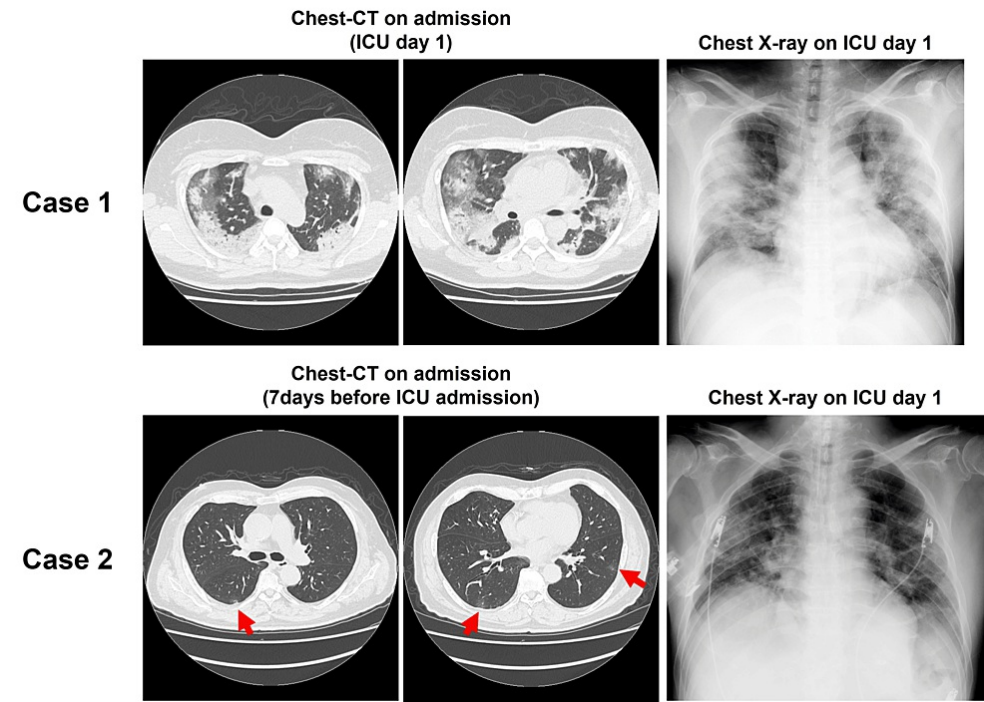
The images of chest computed tomography (CT) on admission and chest X-ray on ICU day 1 In the chest-CT images of case 2, red arrows indicate bilateral and multiple ground-glass opacities.

**Figure 2:**
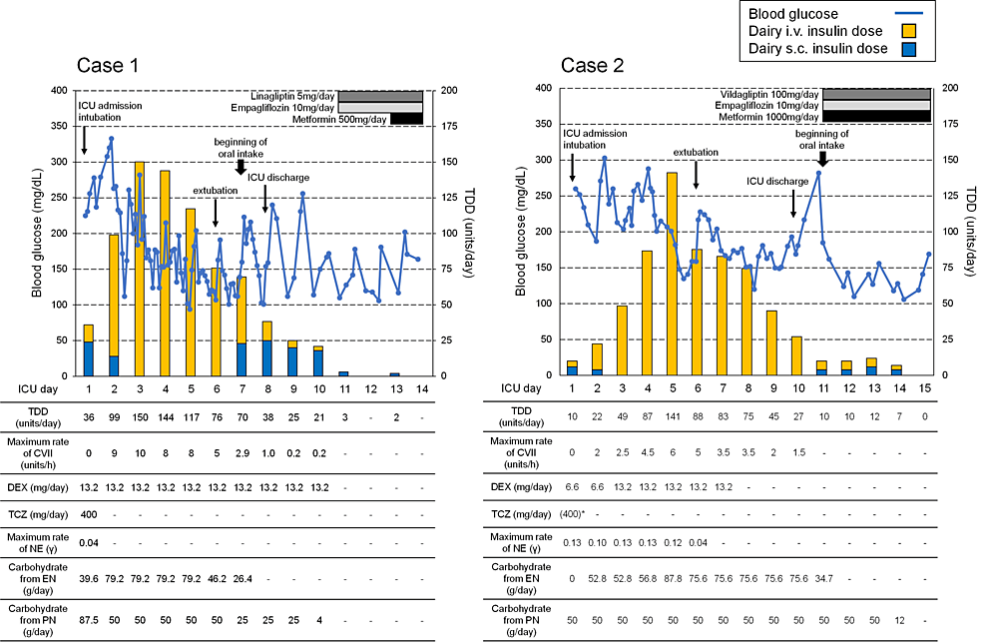
The clinical course of the present cases after ICU admission. * TCZ was administered one day before ICU admission in Case 2. TDD: total daily insulin dose; CVII: continuous venous insulin infusion; DEX: dexamethasone; TCZ: tocilizumab; NE: norepinephrine; EN: enteral nutrition; PN: parenteral nutrition

Case 1 is a 50-year-old Japanese woman whose BMI was 32.4 kg/m2 (weight 82 kg, height 159 cm). She was seeing her family doctor for hypertension. She had been screened for diabetes about three years ago, but no diabetes had been noted. She had never been given the SARS-CoV-2 vaccination. She underwent the SARS-CoV-2 polymerase chain reaction (PCR) test from nasal swabs because her family had been diagnosed with COVID-19, and the result was negative, and she was asymptomatic. Ten days later, she visited a public health center for fever and dyspnea and was transferred to our hospital. Her vital signs were a blood pressure (BP) of 149/87 mmHg, pulse rate (PR) of 96 /min, regular, and a body temperature (BT) of 36.2 °C. She had tachypnea, and her oxygen saturation (SpO2) was 97% on 12 L/min oxygen with a non-rebreather mask. Her HbA1c level was 9.1%, and she was diagnosed with T2DM for the first time. The SARS-CoV-2 PCR test result was positive. Chest X-rays and chest computed tomography (CT) showed bilateral and multiple ground-glass opacities and consolidation (Figure 1). She was diagnosed with COVID-19 pneumonia and admitted to the isolation ward, and then oxygen inhalation with a high-flow nasal cannula (HFNC) was initiated. She received 400 mg of tocilizumab, an IL-6 receptor antibody, and was started on 13.2 mg/day of dexamethasone and 3 g/day of meropenem. Her blood glucose level was 225 mg/dL, and combination therapy with subcutaneous basal insulin and a sliding scale regimen using subcutaneous short-acting insulin were initiated. On the first day of hospitalization, her respiratory status rapidly deteriorated, and she was admitted to the intensive care unit (ICU). Laboratory data on ICU admission are presented in Table 1. She was intubated, and low tidal volume ventilation in the intermittent prone position was initiated. After admission to the ICU, her BP decreased, and norepinephrine administration was started. On hospital day 2 (ICU day 2), because her blood glucose level was above 300 mg/dl despite frequent subcutaneous insulin injections, a continuous intravenous insulin infusion (CVII) was initiated (Figure 2). On hospital day 3 (ICU day 3), an insulin infusion rate of 10 units/h of maximum insulin was required, and the total daily insulin dose (TDD) reached 150 units/day (1.83 units/kg/day). Although her respiratory status gradually improved the next day (ICU day 4), a similar amount (144 units/day) of insulin was still required. From hospital day 5 (ICU day 5), her blood glucose level gradually declined, the insulin dose decreased, and oxygenation improved further. She was extubated on hospital day 6 (ICU day 6), and the TDD was reduced to half of the peak.

**Table 1:** The laboratory data of present cases at ICU admission. WBC: count of white blood cells; RBC: count of red blood cells; Hb: hemoglobin; Hct: hematocrit; PLT: count of platelets; PT: prothrombin time; APTT: activated partial thromboplastin time; FDP: fibrin degradation products; HFNC: high flow nasal canula; AST: aspartate aminotransferase; ALT: alanine aminotransferase; LDH: lactate dehydrogenase; ALP: alkaline phosphatase; γ-GTP: gamma glutamyl transferase; BUN: blood urea nitrogen; TP: total protein; CRP: C-reactive protein; n.m.: not measured

	Case 1	Case 2	Reference range	Unit
< Complete blood count >				
WBC	8,180	13,130	3,300 to 8,600	cells/µL
Neutrophils	85.7	89.3	40 to 72	%
Lymphocytes	9.9	4.5	25 to 47	%
Monocytes	4.2	6.0	3 to 7	%
Eosinophils	0.0	0.0	1 to 5	%
Basophils	0.2	0.2	0 to 1	%
RBC	4.71×10^6^	4.78×10^6^	3.86 to 4.92 ×10^6^	cells/µL
Hb	14.3	14.9	11.6 to 14.8	g/dL
Hct	41.3	42.0	35.1 to 44.4	%
PLT	152×10^3^	141×10^3^	15.8 to 34.8 ×10^3^	cells/µL
< Coagulation fibrinolysis examination >				
PT	83.7	92.8	70 to 140	%
APTT	38.3	32.8	26 to 38	seconds
Fibrinogen	597	515	170 to 410	mg/dL
FDP	3.6	< 2.5	0 to 5	µg/mL
D-dimer	1.5	0.6	0 to 1	µg/mL
< Arterial blood gas analysis >	(HFNC 50 L/min, FiO2 0.9)	(HFNC 30 L/min, FiO2 0.5)		
pH	7.365	7.354	7.35 to 7.45	
pO2	67.5	73.0	83 to 108	mmHg
PCO2	36.8	36.2	32 to 48	mmHg
HCO3^-^	20.5	20.4	21 to 28	mmol/L
< Biochemistry >				
AST	51	31	13 to 30	units/L
ALT	46	24	7 to 23	units/L
LDH	514	430	124 to 222	units/L
ALP	61	165	38 to 113	units/L
γ-GTP	69	27	9 to 32	units/L
BUN	11.9	33.4	8 to 20	mg/dL
Creatinine	0.66	0.74	0.46 to 0.79	mg/dL
Na	142	134	138 to 145	mmol/L
K	3.9	5.4	3.6 to 4.8	mmol/L
Cl	106	100	101 to 108	mmol/L
TP	5.9	5.4	6.6 to 8.1	g/dL
Albumin	2.9	3.5	4.1 to 5.1	mg/dL
Ferritin	1950.3	n.m.	4.6 to 204	ng/mL
Procalcitonin	0.28	0.06	< 0.49	ng/mL
CRP	260	74.3	< 0.14	mg/L
Glucose	185	257	73 to 109	mg/dL
HbA1c	9.1	8.8	4.9 to 6.0	%

Along with discontinuing tube feeding and beginning oral intake, CVII was tapered, and subcutaneous insulin injections were started on hospital day 7 (ICU day 7). Insulin doses were decreased by approximately one-fourth of the peak, and she was discharged from the ICU on hospital day 8. After termination of dexamethasone and infusion, linagliptin 5 mg/day and empagliflozin 10 mg/day were started, and intensive insulin therapy was discontinued on hospital day 11. After initiation of metformin 500 mg/day, she was discharged on hospital day 14 with oral antidiabetic agents alone.

Case 2

Case 2 is a 67-year-old Japanese man whose BMI was 28.7 kg/m2 (weight 78 kg, height 165 cm). He had a history of myocardial infarction and received prescriptions for hypertension, dyslipidemia, and T2DM. He was taking oral antihyperglycemic drugs, such as vildagliptin 100 mg/day, empagliflozin 10 mg/day, and metformin 1000 mg/day. He had never received SARS-CoV-2 vaccination. He visited our hospital with a cough for four days and sore throat and joint pain for two days. His vital signs were a BP of 123/78 mmHg, PR of 78/min, regular, and a BT of 36.4°C. He had tachypnea, and his SpO2 was 94% on room air. His HbA1c was 8.8%. The SARS-CoV-2 PCR test result was positive, and ground-glass opacities were found in the bilateral inferior lobes by chest CT (Figure 1). He was diagnosed with COVID-19 pneumonia and admitted to the isolation ward. After admission, fever and respiratory failure developed despite initiating ciclesonide inhalation and favipiravir administration, and oxygen via nasal cannula and 6 mg dexamethasone were initiated on hospital day four. On hospital day seven, 400 mg of tocilizumab was administered because he required more oxygen to maintain SpO2, and his COVID-19 pneumonia was considered to be exacerbated. Chest X-ray on hospital day eight (ICU day 1) showed bilateral and multiple ground-glass opacities and consolidation (Figure 1). As his respiratory condition rapidly worsened despite oxygenation with HFNC, he was admitted to the ICU and intubated with low tidal volume ventilation in the intermittent prone position. The laboratory data on ICU admissions are presented in Table 1. On the same day, his blood pressure declined, and norepinephrine administration was initiated. Because his blood glucose level exceeded 300 mg/dL despite frequent subcutaneous insulin injections, CVII was initiated (Figure 2). On hospital day 12 (ICU day 5), his TDD increased and peaked at 141 units/day (1.81 units/kg/day) (Figure 2). After that, his respiratory state gradually improved, and he was extubated on hospital day 13 (ICU day 6). His insulin requirements had decreased to approximately half their peak at the time. After the termination of dexamethasone administration, the insulin dose was further decreased. The next day after discharge from the ICU, CVII was discontinued, and subcutaneous insulin injections of vildagliptin 100 mg/day, empagliflozin 10 mg/day, and metformin 1000 mg/day were initiated along with the beginning of oral intake. He was discharged with antidiabetic agents alone on hospital day 36.

## Discussion

SARS-CoV-2 infection induces insulin resistance by producing reactive oxygen species, upregulating IL-6, and activating the renin-angiotensin-aldosterone system [5]. Inflammation associated with SARS-CoV-2 infection may also induce insulin resistance, and it has been reported that insulin requirements in COVID-19 patients are in parallel with the levels of inflammatory markers [9,11]. Moreover, a recent study has shown that SARS-CoV-2 directly infects human pancreatic beta cells, decreases glucose-stimulated insulin secretion, and induces apoptosis [6]. These mechanisms exacerbate glucose metabolism during COVID-19 and may lead to the development of new diabetes mellitus [5,8,12]. Furthermore, clinical approaches for patients with severe COVID-19 exacerbate their glucose metabolism. First, corticosteroids, a common drug used in hospitalized COVID-19 patients requiring oxygen therapy, promote glycogenesis and exacerbate glucose metabolism. Second, the enteral formula for patients with T2DM often causes an acute elevation of plasma glucose levels. Finally, administration of vasopressors or parenteral nutrition may also induce hyperglycemia in critically ill patients. In patients with severe COVID-19, these factors aggravate glucose metabolism, and the complex interplay of these factors can lead to incredible difficulty in glycemic control.

Previous reports have indicated that a high insulin dosage is required for glycemic control in COVID-19 patients with diabetes mellitus [8,9]. In Table 2, we review the characteristics of the present cases and those of previous cases with severe COVID-19 and T2DM whose TDD is available. Wu et al. reported eight cases of severe COVID-19 with mean peak insulin requirements of 201 units/day (2.2 units/kg/day) [9]. In this report, one patient had been treated with extracorporeal membrane oxygenation (ECMO), and six patients had required vasopressor administration. Jornayvaz et al. described a 57-year-old man with T2DM and obesity (BMI 33.5 kg/m2) who required over 1,000 units/day (10 units/kg/day) of intravenous insulin at the peak [13]. This patient required MV and vasopressor therapy. Seggelke et al. reported a Caucasian patient who required 353.6 units/day (2.8 units/kg/day) of peak TDD [14], and this patient had required ECMO, vasopressor therapy, and continuous renal replacement therapy. Affinati et al. showed two cases that required approximately 600-800 units/day (5 units/kg/day and 6.45 units/kg/day) of insulin at the peak [15]. In these cases, one had developed diabetic ketoacidosis on admission, and the other had required ECMO. Satomura et al. described a lean woman (BMI 20.9 kg/m2) who required 150 units/day (2.63 units/kg/day), resembling subcutaneous insulin resistance syndrome [16]. She had needed ECMO and vasopressors for respiratory and circulatory management. On the other hand, in the present cases, the maximum daily insulin requirements were 150 units/day (1.83 units/kg/day) and 141 units/day (1.81 units/kg/day), respectively, less than those of the previous reports mentioned above. However, both cases required MV and vasopressor administration [9,13-15].

**Table 2:** The literature review of previous reports, which describe details of insulin requirements and the related factors in severe COVID-19 patients with type 2 diabetes, compares the present cases. TDD: total daily insulin dose; M: male; F: female; NA: not available; HC: hydrocortisone; mPSL: methylprednisolone; PSL: prednisolone; DEX: dexamethasone; SAR: sarilumab; TCZ: tocilizumab; ECMO: extracorporeal membrane oxygenation; MV: mechanical ventilation; HFNC: high flow of nasal canula *: 6/8 cases had required vasopressor administration. †: 8/8 cases had required MV, and 1/8 cases had required ECMO.

	Age	Gender	Ethnicity	BMI (kg/m^2^)	Maximum TDD (units/kg/day)	Corticosteroid dose at the day most insulin was required (mg/day)	Carbohydrate dose at the day most insulin was required (g/kg/day)	Vasopressor use	IL-6 receptor antibody	Peak CRP (mg/L)	Respiratory support	HbA1c at the admission (%)
Wu L et al. [9] case 1	65	M	NA	27.6	1.10	-	NA	*	-	296	†	7.2
Wu L et al. [9] case 2	39	F	NA	32.1	1.40	-	NA	*	-	334	†	11.9
Wu L et al. [9] case 3	58	M	NA	43.4	1.30	-	NA	*	-	234	†	11.1
Wu L et al. [9] case 4	59	M	NA	35.9	2.30	-	NA	*	-	207	†	8.0
Wu L et al. [9] case 5	71	M	NA	32.5	4.70	-	NA	*	-	214	†	9.9
Wu L et al. [9] case 6	36	F	NA	29.6	3.30	-	NA	*	-	302	†	12.4
Wu L et al. [9] case 7	57	F	NA	35.8	0.890	-	NA	*	-	203	†	6.0
Wu L et al. [9] case 8	55	F	NA	30.9	2.30	-	NA	*	-	440	†	7.7
Jornayvaz FR et al. [13]	57	M	NA	33.5	10.5	HC 150 mg	1.43	+	-	300	MV	6.1
Seggelke SA et al. [14]	59	M	White	38.5	2.81	-	2.80	+	-	456	ECMO	9.0
Affinati AH et al. [15] case 1	41	F	African-American	44	5.00	-	NA	NA	-	NA	HFNC	11.6
Affinati AH et al. [15] case 2	47	F	African-American	39	6.45	mPSL 1mg/kg/day	NA	NA	SAR	NA	ECMO	7.3
Satomura A et al. [16]	52	F	NA	20.9	2.63	PSL 60 mg	NA	+	-	180	ECMO	8.3
Present case 1	50	F	Japanese	32.4	1.83	DEX 13.2 mg	1.68	+	TCZ	260	MV	9.1
Present case 2	67	M	Japanese	28.7	1.81	DEX 13.2 mg	1.66	+	TCZ	74.3	MV	8.8

We considered why our patients achieved glycemic control with even lower insulin requirements than previously reported and examined differences in the severity of inflammation, carbohydrate dosage, and corticosteroid dosage. Wu et al. showed an association between insulin requirements and changes in CRP levels in eight patients [9]. In our cases, the peak CRP in Case 1 was comparable to that in previous cases, while that of Case 2 was considerably lower, and the peak CRP value seemed to be unrelated to the maximum TDD (Table 2). Information on carbohydrate dosage was available in only two cases in these previous reports (Table 2). On the day with the highest insulin dose, the case of Seggelke et al. required 1.56 times more insulin with 1.5 times more carbohydrates than our cases [14]. However, another case reported by Jornayvaz et al. required over five times more insulin, although the amount of carbohydrates was comparable to ours [13]. It is, therefore, not plausible that the amount of carbohydrates was the cause of the low maximum insulin dosage in our cases. Corticosteroids were used in only three of the previous reports we reviewed (Table 2). Considering the glucocorticoid effect of dexamethasone, the impact of corticosteroids in our cases does not appear to be smaller than that of other cases.

We made two speculations regarding the other reasons for the low insulin requirements in our cases. First, differences in insulin sensitivity by ethnicity might have been associated with our cases’ comparatively low insulin requirements. T2DM in East Asians is characterized by beta-cell dysfunction rather than insulin resistance [10,17], and a previous meta-analysis of 74 study cohorts showed that the insulin sensitivity index of East Asians was higher than that of Caucasians [18]. Considering these findings and the fact that our patients’ maximal insulin requirements were lower than those of previous cases, despite the absence of apparent differences in BMI, this patient's insulin sensitivity may have been higher before the onset of COVID-19. The only case reported by Satomura et al. was from East Asia, like our cases, but required high insulin doses, probably because of an unusual condition resembling insulin subcutaneous resistance syndrome [16]. Second, the administration of tocilizumab, an IL-6 receptor monoclonal antibody, might have affected insulin requirements. In physiological conditions, IL-6, a pro-inflammatory cytokine from muscle or adipose tissues, mediates beneficial effects for glucose metabolism via enhancing glucagon-like peptide-1 production [19]. On the other hand, since IL-6 induces insulin resistance and is involved in the formation of a cytokine storm in COVID-19 [5,20], the suppression of IL-6 activity by tocilizumab might have reduced insulin resistance via the amelioration of inflammation and cytokine release syndrome. In addition, because IL-6 levels decrease by rectifying hyperglycemia in COVID-19 patients [21], tocilizumab administration also might have improved the vicious cycle between hyperglycemia and IL-6 and influenced insulin requirements. To the best of our knowledge, this is one of the few reports describing the possibility that ethnic differences or the administration of IL-6 inhibitors may affect insulin requirements in severe COVID-19 patients with diabetes.

We have discussed above the causes of the lower insulin requirements in our cases compared to previous reports, but there are some limitations. First, none of our cases were vaccinated, and we were not able to examine the influence of vaccination. Second, since we did not examine the SARS-CoV-2 variant, we could not discuss the impact of differences in variants. Third, these cases were treated at a single institution, and the influence of inter-institutional differences in treatment strategies cannot be excluded. Finally, in the previous cases we reviewed in this study, IL-6 suppression therapy was used in only one case, and information such as ethnicity or carbohydrate dosages was not available for some of them. A large-scale study is needed to determine the differences in insulin requirements according to the ethnic background or the use of IL-6 inhibitors in severe COVID-19 patients with T2DM.

Evidence is accumulating that glycemic control is essential for hospitalized patients with COVID-19, such as patients with uncontrolled hyperglycemia during hospitalization, with or without diabetes, who have a higher risk of progressing to critical illness and increased in-hospital mortality [21-25]. Better glucose control is crucial in preventing poor outcomes in patients with COVID-19, and identifying insulin requirements and factors affecting them is clinically important.

## Conclusions

Ethnic differences in insulin sensitivity or the administration of IL-6 receptor antibodies may affect insulin requirements in patients with severe COVID-19. Further knowledge about the details of insulin requirements in patients with T2DM developing severe COVID-19, including these factors, is helpful for early optimization of insulin dosage, avoiding hypoglycemia, and improving their prognosis.
